# Emerging Targets in Pituitary Adenomas: Role of the CXCL12/CXCR4-R7 System

**DOI:** 10.1155/2014/753524

**Published:** 2014-11-17

**Authors:** Federica Barbieri, Stefano Thellung, Roberto Würth, Federico Gatto, Alessandro Corsaro, Valentina Villa, Mario Nizzari, Manuela Albertelli, Diego Ferone, Tullio Florio

**Affiliations:** Department of Internal Medicine and Medical Specialties and Center of Excellence for Biomedical Research (CEBR), University of Genova, Viale Benedetto XV, 2-16132 Genova, Italy

## Abstract

Chemokines are chemotactic regulators of immune surveillance in physiological and pathological conditions such as inflammation, infection, and cancer. Several chemokines and cognate receptors are constitutively expressed in the central nervous system, not only in glial and endothelial cells but also in neurons, controlling neurogenesis, neurite outgrowth, and axonal guidance during development. In particular, the chemokine CXCL12 and its receptors, CXCR4 and CXCR7, form a functional network that controls plasticity in different brain areas, influencing neurotransmission, neuromodulation, and cell migration, and the dysregulation of this chemokinergic axis is involved in several neurodegenerative, neuroinflammatory, and malignant diseases. CXCR4 primarily mediates the transduction of proliferative signals, while CXCR7 seems to be mainly responsible for scavenging CXCL12. Importantly, the multiple intracellular signalling generated by CXCL12 interaction with its receptors influences hypothalamic modulation of neuroendocrine functions, although a direct modulation of pituitary functioning *via* autocrine/paracrine mechanisms was also reported. Both CXCL12 and CXCR4 are constitutively overexpressed in pituitary adenomas and their signalling induces cell survival and proliferation, as well as hormonal hypersecretion. In this review we focus on the physiological and pathological functions of immune-related cyto- and chemokines, mainly focusing on the CXCL12/CXCR4-7 axis, and their role in pituitary tumorigenesis. Accordingly, we discuss the potential targeting of CXCR4 as novel pharmacological approach for pituitary adenomas.

## 1. Background

Chemokines (CKs) are low molecular weight chemoattractant peptides, belonging to the cytokine family [1]. Differently from interleukins, CKs act* via* G protein-coupled receptors (GPCRs), controlling cell migration and trafficking throughout the body, during immune response [2] and development [3, 4]. CKs are also critical mediators of several physiological mechanisms such as wound-healing and tissue homeostasis [3, 5]; moreover, CKs are expressed in the central nervous system (CNS) [6, 7] where they not only act as mediators of development, intercellular communication, and inflammatory processes but also function as neurotransmitters or neuromodulators, mainly involved in neuroendocrine regulations [8]. Recently, it has been shown that CKs play a relevant role in tumorigenesis, neoangiogenesis, tumor progression, and metastasization [9, 10]. Evidence for autocrine/paracrine regulatory mechanisms in different normal and cancer cell types, driven by chemokine/receptors interaction on the same or a nearby cell, supports the potential role of CKs in the control of physiological or tumoral endocrine functions. In particular, the chemokine (C-X-C motif) ligand 12 (CXCL12) and its receptors, CXCR4 and CXCR7, have been involved in cancer cell proliferation, migration, and invasion [11–13].

Anterior pituitary adenomas account for approximately 15% of primary intracranial tumors. They are classified by size (microadenoma, <10 mm or macroadenoma, >10 mm) and on the basis of their ability to produce hormones, as secreting or functioning tumors (about 50% of adenomas) or as clinically nonfunctioning pituitary adenomas (NFPA) that do not release hormones or, more often, secrete clinically nonrelevant (i.e., gonadotropins) or nonbioactive hormones (*α*-subunit of glycoproteic hormones) [14, 15]. Almost all pituitary tumors display a benign clinical course being slow growing and show low incidence of metastasis; however, they are frequently associated with high morbidity and mortality due to mass-related effects and paraneoplastic syndromes related to hormone hypersecretion. Functioning pituitary adenoma leads to hypersecretion of hormones that results in classic clinical syndromes, mainly acromegaly (overproduction of GH), hyperprolactinemia (excess of PRL), and Cushing's disease (overproduction of ACTH) and, more rarely, secondary hyperthyroidism (increased TSH secretion). These tumors can be monohormonal or plurihormonal. NFPAs do not secrete sufficient hormones (mainly FSH or LH) to be detectable in the blood or to cause hormonal manifestations; in other cases only biologically inactive *α*-subunit is released and more rarely they are classified as true nonsecreting adenomas. Importantly, all pituitary adenomas may induce hypopituitarism and neurological symptoms (for example compression of the optic chiasma) due to mass effect [16, 17]. Clinical relevance and recent advances in the comprehension of their molecular pathogenesis suggest that pituitary adenomas should be considered a more critical disease than a benign endocrine pathology. Thus, a deeper evaluation of the mechanisms at the basis of their tumorigenesis and better prognostic markers to identify tumors with a high risk of recurrence are most awaited to improve pituitary adenoma clinical outcome.

This review will focus on the diverse role of CXC chemokines and their receptors in normal pituitary cell functions and pituitary tumor development and progression, summarize recent progress in CXCR7 functions, and discuss the present issues and future perspectives.

## 2. Chemokine Classification and Receptor Interactions 

Human CK system includes approximately 50 peptides and 22 GPCRs. CKs are classified, according to the number and spacing of the first two cysteine residues of a conserved cysteine motif, into four groups: (1) CXC (with a single nonconserved amino acid residue -X- between the first N-terminal C residues: CXCL1-17); (2) CC (two adjacent cysteine residues: CCL1-28); (3) XC (only one N-terminal cysteine: XCL1-2); (4) CX3C (three nonconserved amino acid residues separating the N-terminal C residues: CX3CL1) [18, 19].

CK receptors are typical GPCRs, all of them signaling through heterotrimeric G proteins (G*α*, and G*β*-*γ* subunits), with the remarkable exception of CXCR7 which is exclusively biased towards *β*-arrestin-mediated signaling [20]. Upon ligand binding, many CK receptors may form homo- and heterodimers that activate distinct intracellular signaling pathways from individual receptors [21]. The classification of CK receptors is based on the class of their ligands (e.g., CXC ligands bind CXC receptors).

CKs may also bind a small group of so-called “atypical chemokine receptors” (ACKRs), which are unable to initiate downstream conventional G-protein-dependent signaling, resulting, from a functional point of view, in the inability to induce directional cell migration [22]. These receptors bind distinct and complementary range of CKs and likely control CK networks during development and physiopathological processes by scavenging CKs. ACKRs, including Duffy antigen receptor for chemokines (DARC, ACKR1), C6 (ACKR2), CXCR7 (ACKR3), and CCX-CKR1 (ACKR4) (for classification and nomenclature see [23]), were proposed to serve as decoy receptors to scavenge inflammatory CKs from the extracellular microenvironment, inhibiting their signaling [22]. Indeed, recent evidence supports the ability of ACKRs to transport, internalize, and degrade CKs, leading to the formation of CK gradients in normal and cancer tissues, responsible for the functional modulation of their signaling.

CKs belonging to the CXC family can be further grouped according to the presence or absence of a the tripeptide motif Glu-Leu-Arg (ELR) preceding the CXC domain (ELR^+^ or ELR^−^), which affects receptor binding specificity and biological effects. Notably, ELR^−^ CXC chemokines (i.e., CXCL9, -10 and -11) are interferon *γ*-inducible and act as potent angiostatic factors to impair angiogenic stimuli induced by growth factors. Conversely, ELR^+^ CKs (CXCL1, -2, -3, -5, -6, -7, -8) are proangiogenic [24, 25]. CXCL12 (previously known as stromal cell-derived factor-1, SDF-1) is an exception to this characterization, since it is ELR^−^ but mediates tumor-promoting angiogenesis* via* its receptor CXCR4 [9, 24, 26].

Common features shared by all CKs are pleiotropism, promiscuity, and redundancy, with a single CK able to bind several receptors, whereas multiple CKs bind the same receptor resulting in the same functional outcome [27].

Upon ligand binding, CK receptors undergo conformational change that activates the G*α* subunits sensitive to* Bordetella pertussis *toxin (PTX). Their activation dissociates the GTP-bound G*α* subunit from the G*β*-*γ* dimer, and both these active components trigger intracellular signals, such as activation of phospholipase C (PLC)/inositol triphosphate (IP3)-Ca^2+^/diacyl glycerol (DAG)/protein kinase C (PKC) and inhibition of adenylyl cyclase (AC)-cAMP/protein kinase A (PKA). Moreover, these receptors control the activity of different kinases, including extracellular regulated kinases (ERK1/2), c-Jun N-terminal kinase (JNK), p38, phosphatidyl inositol 3 kinase (PI3K)-Akt, and the focal adhesion kinase (FAK).

The distinct transductional cascades regulated by CK receptors mainly depend on the G*α* subfamily which they activate: G*α*i inhibits AC but also activates tyrosine kinases of the Src family, favoring signal integration; G*α*q increases PLC*β* activity [12], to cleave PIP2 to form DAG and IP3. In turn, DAG activates PKC, whereas IP3 binds specific receptors on the endoplasmic reticulum inducing Ca^2+^ release from intracellular stores. Finally, G*α*12 controls the activity of the small G protein RhoA,* via* Rho-GEF. On the other hand, CK receptor activation of G*β* *γ* subunits results in the activation of PI3K leading, through the phosphoinositide-dependent kinase 1 and 2 (PDK1-2), to Akt phosphorylation and subsequent activation of its downstream signal proteins such as glycogen synthase kinase 3 (GSK3), mammalian target of rapamycin (mTOR), and FAK, which control migration in different types of normal and tumor cells [28].

After stimulatory responses, the inactivation of CK receptor signaling occurs after the hydrolysis of GTP to GDP by the intrinsic GTPase activity of G*α* subunit, followed by its reassociation with G*β*/*γ* in an inactive complex. Moreover, receptor desensitization, internalization, and lysosomal degradation are mediated by G protein-coupled receptor kinases (GRKs) and arrestins [29].

## 3. Physiological Functions of CKs: Focus on the CXCL12/CXCR4-R7 Axis in the CNS

CKs are constitutively secreted by leukocytes, fibroblasts, endothelial, and epithelial cells to mediate cell activation, trafficking, and homing [5, 30]. Beside their basal expression, most CKs are highly induced during inflammatory or infective processes driving different phases of immune response* via* a CK gradient which directs leukocyte recruitment to the site of inflammation. Furthermore, CKs directly activate specialized effector lymphocytes during the different steps of immune response, for example, CXCL8 (formerly named IL8) recruits neutrophils, basophils, and eosinophils expressing its receptors, CXCR1 and CXCR2 [2]. Adaptive immune responses are mediated by CKs (CXCL9-L10-L11) secreted by macrophages activated by INF-*γ* released by natural killer and T helper 1 (Th1) cells that express CXCR3, the receptor for CXCL9-L11 [31], thus amplifying leukocyte recruitment and, finally, inflammation.

CKs also play a key role in embryogenesis, organogenesis, angiogenesis, and germ cell migration, especially during neural development [5]. The constitutive expression of CKs and their receptors in adult normal brain was initially identified in the immune-like competent cell populations such as microglia and astrocytes. The subsequent detection of their expression in neurons [32–34] broadened CK role as neuromodulators/neurotransmitters in neurological processes such as thermoregulation, pain perception, and stress conditions, as well as in pituitary functions.

Focusing on the CXCL12/CXCR4-R7 network, it exerts a variety of functions in CNS development as well as in mature brain. CXCL12 directs the migration of embryonic and adult stem cells in the developing central and peripheral nervous system [35, 36], controlling the formation of cerebellum, cerebral cortex, hippocampus, and dorsal root and sympathetic ganglia [25, 37, 38]. Postnatally, CXCR4 expression, while downregulated in many brain areas, persists in the hypothalamus where it modulates the hypothalamic-pituitary system and the hypothalamic-pituitary-gonadal axis, in particular, cooperating to the regulation of neuroendocrine and reproductive systems [39–41].

As far as CNS development is concerned, the pivotal role of CXCL12/CXCR4 emerged from studies using knockout mice for either the ligand or the receptor. Both models exhibited a superimposable abnormal neuron migration in the cerebellum, dentate gyrus, and dorsal root ganglia [35, 36, 42, 43]. Furthermore, CXCL12/CXCR4 axis controls migration and homing of Cajal-Retzius cells [44, 45], postmitotic neurons [46], cortical interneurons [40, 47–49], and dopaminergic neurons [50]. CXCL12/CXCR4 regulation of stem cell positioning and migration persists in adults, in the neurogenic niches of brain and in the bone marrow, where hematopoietic progenitors cells are retained by the interaction between ligand and receptor [51] that also promotes their survival and proliferation. Interestingly, a similar homing mechanism has been demonstrated for adult neural progenitor cells (NPCs) or neural stem cells (NSCs) [25].

CXCR7, the second CXCL12 receptor, has a 10-fold higher binding affinity than CXCR4 but also binds CXCL11 (formerly known as IFN-inducible T cell *α* chemoattractant, I-TAC,), which, in turn, interacts with CXCR3 [52]. Presently, the function of CXCR7 is still controversial [53]. CXCR7 does not mediate CXCL12-dependent cell migration [20, 52, 54] and displays atypical signaling pathways, failing to induce intracellular Ca^2+^ mobilization and inhibition of cAMP production, since this receptor does not seem to be coupled to Gi*α*. Based on its ability to rapidly sequester and degrade CXCL12 and thus to suppress CXCR4 activity, CXCR7 was firstly proposed to be a decoy receptor [54–57]; currently, this activity is considered only a part of the possible mechanisms by which CXCR7 modulates cellular functions [22]. Indeed, emerging evidence suggests that CXCR7 can promote cell motility [58–60] and trigger intracellular signals in different human normal and cancer cell types [61–64]. In particular, CXCR7 activates Akt, MAP kinase (MAPK), and JAK/STAT3 cascades, either by direct modulation, through a *β*-arrestin-dependent pathway [20, 65], or after heterodimerization with CXCR4 [59, 66–69]. Preferential signaling through G-proteins or *β*-arrestin is influenced by both CXCR4-CXCR7 dimer formation and the oligomerization state of CXCL12 [70, 71]. CXCR7 was recently shown to activate mTOR in human renal cancer cells through the modulation of ERK1/2 and p38 activities [72], further suggesting that it is a fully signaling receptor although independent from G proteins. However, CXCR7 knockout mice display a lethal phenotype due to a heart valve and vascular defects [73], a very similar scenario observed in mice with targeted disruption of the genes encoding CXCR4 and CXCL12 [67, 74].

In the adult rat brain, CXCR7 is expressed at high levels in vessels, pyramidal cells, and mature dentate gyrus granule cells, overlapping CXCL12 expression pattern [75, 76], and a functional role for CXCR7 in the control of neuronal migration to the subventricular and intermediate zone was suggested [69, 77]. In rat mature neurons and blood vessels, CXCR7 appears to be the preponderant CXCL12 receptor, likely contributing to CXCL12-dependent neuronal development [75]. Moreover, CXCR7 acts as scavenger on brain microvessel endothelium [78] and it is essential for inflammatory leukocytes to infiltrate the CNS [79]. CXCR7 is also expressed in neural tube and brain of mice embryos. In rat cortex, CXCR7 is localized in GABAergic neuron precursors, and Cajal-Retzius cells and, unlike CXCR4, it has been identified in neurons forming the cortical plate and in the developing dentate gyrus and cerebellar external germinal layer [75, 80].

Migrating immature cortical interneurons co-express CXCR4 (membrane surface expression) and CXCR7 (intracellular expression, mainly endosomes) [77]. CXCR7 rapidly recycles from membrane to intracellular pools of interneurons, and its trafficking mediates CXCL12 endocytosis. It is essential for the regulation of interneuron migration in the developing cerebral cortex since its removal causes an increase in extracellular CXCL12 content, which favors its binding to CXCR4 and consequently induces the endocytosis and degradation of CXCR4. Thus, CXCR7 regulates CXCR4 expression and likely controls CXCL12 signaling to drive successful migration in the developing cerebral cortex [77]. In addition, since CXCR7^−/−^ and CXCR4^−/−^ mutant mice displayed opposite defects in interneuron motility and positioning, CXCR4 and CXCR7 were proposed to have distinct roles and signal transduction to regulate interneuron movement [69]. This fine tuning of CXCL12 response induced by CXCR7 occurs either directly modulating *β*-arrestin-mediated signaling cascades or scavenging local CXCL12 availability [81].

Deletion of one of the CXCL12 receptors is sufficient to generate a migration phenotype that corresponds to the CXCL12-deficient pathway and interfering with the CXCL12-scavenging activity of CXCR7 causes loss of CXCR4 function [81]. For example, during development, CXCL12 regulates the migration of gonadotropin-releasing hormone (GnRH) neurons, through CXCR4-mediated activation of the GIRK channel [82], but this effect is modulated by CXCR7 which controls CXCL12 content availability acting as a scavenger along the migratory path [83].

The relevance of CXCL12 and CXCR4-R7 system in CNS ontogeny and functions is even more crucial in the view of their expression in both embryonic and adult brain stem cells, a subset of undifferentiated cells characterized by self-renewal through asymmetric division, differentiation into multiple lineages, and constant proliferation that in adults acts in tissue maintenance and repair [84]. The role of CXCL12 and CXCR4 in stemness maintenance has gained much attention also in the neuroendocrinology field due to the proposed role of stem cells in pituitary plasticity [84, 85]. Both CXCL12 and CXCR4 are expressed in different anterior pituitary cell subtypes, as well as in nonhormonal cell types [86–88]. The chemotactic activity of this CK could be also relevant in folliculostellate (FS) cell, non-FS nestin^+^ cell, and stem cell migration [86, 89]. Therefore, understanding the CK-dependent mechanisms associated with candidate stem cells within pituitary might help to clarify their activity in development or in normal mature hormone-producing and tumor pituitary cells [84, 90, 91].

The stem cell concept applied to cancer has radically changed the research approach to tumorigenesis and treatment, since the subset of cancer cells, namely, cancer stem cells (CSCs), seems responsible for tumor initiation, metastasis, and resistance to therapy [92]. Although, at present, all factors and signals that regulate CSCs are not completely clarified, accumulating evidence suggests a key role of the CXCL12/CXCR4 axis in CSC maintenance and growth [62, 93]. Moreover, interactions between CSCs and tumor microenvironment through secreted CKs (e.g., CXCL12) [13], possibly occurring also in pituitary adenomas, may act as chemoattractant to recruit fibroblasts, endothelial, mesenchymal, and inflammatory cells to the tumor,* via* CXCR4.

## 4. Chemokine Functions in Normal Pituitary 

Through the release of growth factors (bFGF, EGF, and VEGF), cyto/chemokines, and neuroendocrine proteins (steroid hormones, prolactin, growth hormone, ghrelin, erythropoietin, catecholamines, etc.) neuronal and neuroendocrine pathways regulate fundamental functions within the CNS and its interaction with the immune system [94]. Complex autocrine/paracrine signals through neuropeptides (e.g., EGF and VIP), neurotransmitters, cytokines (IL-1, IL-6), and CKs occur also in pituitary regulation, differently from the classical hypothalamic input and feedback signals from the periphery [95–97].

EGF expression has been observed at all stages of pituitary development and in the adult pituitary, and the EGFR pathway contributes to pituitary physiology and tumorigenesis [98].

IL-1*β* receptors were detected in pituitary cells [99], and their activation inhibits prolactin (PRL) secretion from dispersed rat pituitary cells through the regulation of AC and PLC activities, and Ca^2+^ fluxes [100–103].

IL-6 and its receptors are also expressed in the pituitary gland [104, 105] where their interaction regulates apoptosis and proliferation of endocrine cells* in vitro* [106]. IL-6 is mainly produced by the FS cells and activates a paracrine loop on the hormone-secreting cells [107, 108] regulating ACTH [109], PRL, LH, and GH secretion [110–112]* via* the modulation of AC and PLC activities [113]. Interestingly, IL-6 exerts opposite effects on normal and adenomatous pituitary cells: it is inhibitory for normal anterior pituitary [114] and stimulatory for adenoma cells [107].

IL-18 was also proposed to exert paracrine effects in pig anterior pituitary being the ligand and its receptor expressed by different subsets of GH secreting cells [115].

Finally, interleukins' regulation of the hypothalamic-pituitary-adrenal axis also involves the modulation of vasoactive intestinal peptide- (VIP-) secreting pituitary cells to control, in a paracrine manner, PRL release [116].

More recently, several studies were directed on the role of CKs in pituitary. CKs can affect pituitary hormone secretion* via* the hypothalamic-pituitary axis or autocrine/paracrine regulation. CXCL1 is expressed in the posterior pituitary, in the paraventricular nucleus (PVN) of the hypothalamus and the median eminence [6]. In response to stressful stimuli, this CK is released in the median eminence [117] to reach its receptor (CXCR2) expressed in pituitary cells and induce the release of PRL and GH and the inhibition of LH and FSH secretion [118]. Similarly, CCL2 was identified in both hypothalamus and pituitary [119]. The generation of transgenic rats (S100*β*-GFP rats) that express green fluorescent protein in S100*β*-positive pituitary FS cells in the anterior pituitary [120] led to the characterization of S100*β*-positive cells [86, 121] and transcripts of CXCL10 (IFN-*γ* inducible protein 10 kDa, IP-10) were identified in a subpopulation of these cells. Importantly, CXCR3, the receptor for CXCL10, was shown to be expressed in corticotrophs, suggesting a possible autocrine/paracrine effect of CXCL10, released from FS cells, on ACTH-producing cells [122].

CXCL12/CXCR4 is the major regulatory axis not only connecting the immune and nervous systems, but also playing a role in neuroimmune regulation of the anterior pituitary physiological functions [6].

CXCL12 was detected in both rat pituitary [123] and hypothalamus [124] and its expression in hypothalamic neurons, concomitant with CXCR4 positivity at pituitary level [124], corroborated the hypothesis that this CK could represent a hypothalamic regulatory factor of anterior pituitary function. As a consequence, the chemokinergic regulation of anterior pituitary cells might derive from coordinate activity of CXCL12 originating from both hypothalamic neurons and systemic circulation [95]. A regionalized constitutive expression of CXCL12 was reported in adult rat brain, particularly in arginine vasopressin- (AVP-) expressing neurons [125] where its interaction with CXCR4 leads to modulates induced plasma AVP release* in vivo* [39]. The expression pattern of this chemokine and its receptor in the rat hypothalamo-neurohypophyseal system was further investigated: they colocalize within AVP-expressing neurons in both supraoptic (SON) and paraventricular (PVN) nucleus as well as in dense core vesicles of AVP-positive nerve terminals in the posterior pituitary, showing a similar distribution [126]. Since AVP controls body fluid homeostasis, the interaction between CXCL12 and AVP was studied in AVP-deficient Brattleboro rats that show low expression of both CXCL12 and CXCR4, correlated with AVP protein expression level in SON, PVN, and posterior pituitary. However, since CXCL12 mRNA is increased, it was hypothesized that CXCL12 synthesis is present in these cells but, being costored with AVP, a concomitant massive release of both peptides is responsible for their low content at both hypothalamic and posterior pituitary levels [126]. AVP and CXCL12 expression is dependent on water balance and is centrally regulated, further strengthening the role of CXCL12 in neuroendocrine functions. However, CXCL12 and CXCR4 are also coexpressed in rat pituitary cells [127] and a further autocrine/paracrine regulation of pituitary functioning was hypothesized. Complete colocalization between CXCR4 and GH was reported in normal rat pituitary, suggesting that CXCR4 is a rather specific regulator of somatotroph activity, in rats [128]. Indeed, CXCL12 stimulates GH transcription and secretion in both primary rat anterior pituitary cells and the GH-producing pituitary adenoma cell line, GH3 [128]. Interestingly, rat FS cells also express CXCR4 and secrete CXCL12, which acts as a potent chemoattractant for these cells. The activation of this autocrine loop facilitates the formation of F-actin in FS cells and the subsequent directional extension of their cytoplasmic processes toward other FS cells [86]. CXCL12/CXCR4 interaction induces invasion and interconnection of FS cells to near lobular structures likely forming a circuit that causes or maintains local cellular arrangement in the anterior pituitary [86].

**Figure 1:**
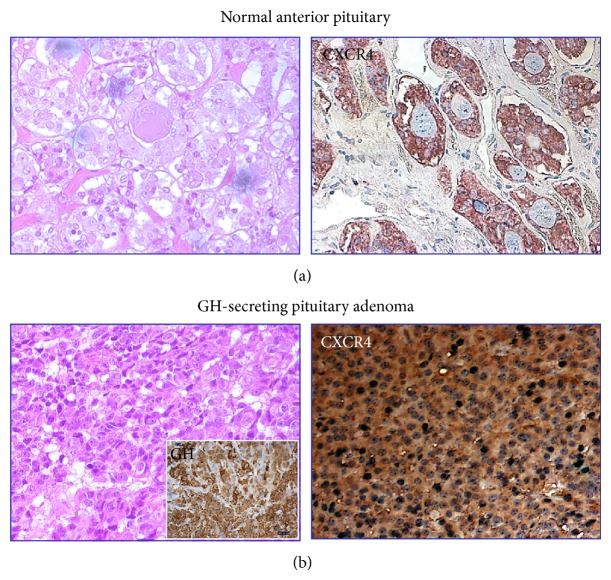
Overexpression of CXCR4 in human pituitary adenomas as compared to normal human adenohypophysis. Immunohistochemical images of human GH-secreting adenoma showing the marked homogeneous positivity for CXCR4 throughout the tissue as compared to scattered staining evidenced in normal anterior pituitary. Hematoxylin and eosin staining and GH-positivity are also depicted. (Original magnification 40x).

**Figure 2:**
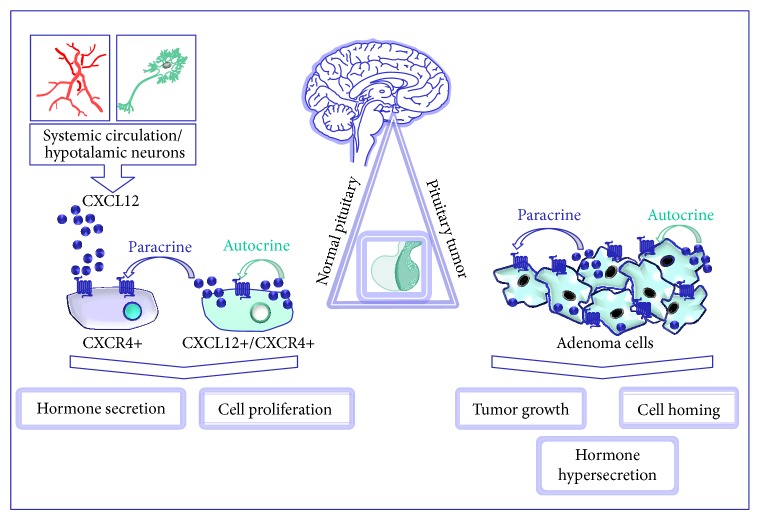
CXCL12/CXCR4 system represents a regulatory pathway for pituitary both in physiological functions and tumorigenesis. CXCL12 originating from hypothalamic neurons or systemic circulation represents a regulatory factor of anterior pituitary function. Autocrine and paracrine mechanisms control proliferative and secretagogue activities in normal pituitary cells expressing either CXCR4 alone or CXCR4 and CXCL12. Overexpression of CXCR4 and its ligand induces autocrine/paracrine proliferation in pituitary tumor cells and likely contributes to adenoma development.

In humans, a slightly different pattern of expression was found in autoptic normal pituitaries. Scattered expression of both CXCR4 and CXCL12 within the anterior lobe was detected by immunohistochemistry, revealing a nonhomogeneous positivity for both proteins throughout the tissue, including large negative areas, others showing few positive cells and rare zones with higher expression [129] (Figure 1). Interestingly, in all these areas, CXCR4 expression resulted largely higher than its ligand, although all the CXCL12-positive cells express CXCR4, as well. CXCR4-expressing cells do not belong to specific secreting cell type, being present in GH, PRL, or ACTH-secreting cells, while no expression was observed in human FS cells. However, some CXCR4-expressing cells do not coexpress any hormones and no colocalization of either CXCR4 or CXCL12 was observed in FS cells; thus it was proposed that CXCL12/CXCR4 system may also label undifferentiated/progenitor cells. Conversely, the rare CXCL12-positive cells were mainly, although not exclusively, corticotrophs [129]. Notably, this CK-receptor pair was undetectable in human posterior pituitary lobe [129], contrarily to what was observed in rats. Thus, from these data it is evident that, in normal pituitary, CXCL12 is secreted by cell subpopulations that, cooperating with hypothalamic factors (including CXCL12 itself), may contribute to paracrine modulation of pituitary functioning (Figure 2). Consequently, alterations of the endocrine regulatory pathways due to upregulation of hypothalamic/pituitary CXCL12/CXCR4 axis might lead to the development of pituitary adenomas [127, 129].

The activity of CXCR7 in normal pituitary deserves further investigation; however its expression in pituitary adenoma tissues [130, 131] suggests possible involvement in pituitary function regulation.

## 5. CXCL12/CXCR4-R7 in Cancer Development and Progression: Autocrine/Paracrine Loops

Beside direct CXCR4-dependent activation of ERK1/2, transactivation of tyrosine kinase receptors is currently a relevant mechanism in tumor cell responses. Mainly, the transactivation of epidermal growth factor receptor (EGFR) family members mediates the mitogenic activity of different CKs in human cancer. A cross-talk between CXCL12 and EGFR and/or HER2/neu phosphorylation was demonstrated in breast and ovarian cancer cells through G protein-dependent activation of kinases of the Src family [132–134]. Moreover, in breast cancer, CXCR4 interacts with the EGFR variant, EGFR vIII, a constitutively active mutant highly expressed in cancer stem cells [135], to regulate invasion* via* p38 MAPK [136]. CXCR4 signaling is negatively regulated by protein-tyrosine phosphatases (PTPs), such as the Src homology-containing protein-tyrosine phosphatase 1 (SHP1) and the SH2 domain-containing inositol 5-phosphatases (SHIP), while SHP2, constitutively associated with CXCR4, potentiates CK signaling [137, 138]. These observations are particularly relevant since they highlight possible direct antagonisms between CXCR4 and somatostatin receptors (SSTR) that are powerful activators of PTPs [139–141]. This antagonistic activity could acquire clinical relevance in light of the fact that SSTR agonists are the main pharmacological tool available for the treatment of pituitary adenomas [142, 143].

The concomitant expression of ligand-receptor pair in the same tumor cells, responsible of autocrine/paracrine activation, is one of the leading causes of clinical aggressive behavior in various cancer types [144, 145]. Exploiting the coexpression of CKs and their receptors, cancer cells and cells of the tumor microenvironment are able to modulate immune response, promote angiogenesis, and sustain proliferation [146]. As previously described, this autocrine mechanism is maximally effective for CXCR4, the most widely expressed CK receptor in human solid and hemopoietic malignancies [28]. The autocrine/paracrine loop of the CXCL12/CXCR4 pair has been deeply investigated in brain tumors in both* in vitro* and* in vivo* models: CXCL12 stimulates proliferation and migration of glioblastoma cells and xenografted tumors inducing ERK1/2 and Akt phosphorylation [147–149]. The mitogenic activity mediated by CXCL12-CXCR4 was also reported in meningioma, in which CXCR4 activation increased DNA synthesis through activation of ERK1/2 in primary cultures and its expression level significantly correlated with Ki-67 proliferation index of the original tumor tissue [150, 151], while CXCR7 was mainly localized in tumor endothelia [152].

Similar CXCR4 tumor-promoting effects were observed in breast carcinoma [80], suggesting CXCL12 as possible autocrine/paracrine growth factor [153]. Interestingly, in breast cancer cells the synthesis and release of CXCL12 is under the control of 17*β*-estradiol contributing to its proliferative effects and mediating,* via *a Src-dependent mechanism, EGFR transactivation [133, 154].

Furthermore, CXCL12 is also indirectly implicated in tumor pathogenesis, acting as chemoattractant for CXCR4-positive cells, directing tumor cell migration [155–157] and controlling invasive and metastatic properties of CXCR4-expressing cancer cells to distant organs [158, 159]. The invasive behavior of cancer cells might indirectly depend on locally released CXCL2 that,* via* an autocrine mechanism, binds to CXCR4 impairing chemotaxis towards CXCL12-producing target organs and metastatic spread [160]. In addition, normal cells forming tumor stroma (i.e., macrophages, lymphocytes, fibroblasts, and endothelial cells) concur to cancer development and progression through CXCL12 secretion. CXCL12 concentration gradient directs cancer cell motility in several tumors [10, 161], including aggressive solid neoplasms (breast [153], colon [162], brain [149] ovarian [163], prostate [161], renal cell [164] and oral squamous cell [165] carcinomas, and melanoma [166]). Importantly, as observed in a rat mammary adenocarcinoma cell line overexpressing both receptors [167], CXCR4 and CXCR7 play opposing roles in breast cancer metastasis: CXCR4 mediates cancer cell invasion allowing cells to follow the CXCL12 gradient generated by metastatic targets whereas CXCR7 favors tumor growth increasing angiogenesis but impairs cell migration scavenging the chemokine.

## 6. A Network Map of Proliferative Signaling in Pituitary Tumor Development

Raf/MEK/ERK and PI3K/Akt pathway dysregulation is a common alteration responsible of tumor initiation and progression. While the pathways are classically activated by growth factors, cross-talks and transactivation mechanisms with neuropeptide-cytokine-CK/GPCRs have been increasingly recognized. This cross-talk activates ERK1/2, which is directly responsible for cell growth and differentiation, depending on the cellular context and represents one of the major proliferative pathways in cancer [168, 169].

The overexpression or constitutive activation of receptors for growth factors, cytokines, and CKs potentiates the activation of Ras/Raf/MEK/ERK pathway also in pituitary adenoma [170]. MAPK phosphorylation is relevant in different pituitary cell types such as AtT20 cells, where it regulates CRH-induced POMC transcription [171], gonadotrophs for GnRH signaling [172], GH-secreting cells for GHRH-dependent cyclin D1 expression [169], and GH4C1 somatotroph cell line in which the Gsp oncogene impairs Ras/ERK1/2-dependent PRL gene regulation [173]. PI3K/AKT/mTOR pathway, activated by a variety of growth factors and hormones, when dysregulated, leads to aberrant growth of pituitary adenoma cells. Akt is overexpressed and hyperphosphorylated in NFPA [174], in a mouse model of TSH-oma [175], and in GH3 cells in which the inhibition of PI3K/Akt signaling by octreotide increases the expression of the tumor suppressor gene Zac1 [176, 177].

Similarly to cell proliferation, survival mechanisms also sustain pituitary development and tumorigenesis: in particular the balance of pro- and antiapoptotic factors [178], physiologically contributing to normal pituitary cell plasticity, when unbalanced, favors pituitary cell transformation [179]. For example, in pituitary adenomas, antiapoptotic mediators, such as the bcl-2 protein family, are upregulated [180], while Fas, a major apoptotic factor in different cell types, activates apoptosis in both normal rat lactotrophs and somatotrophs [181] and in pituitary adenoma cell lines [182]. Several pituitary-related genes may exert a role in apoptosis of secretory pituitary cells, as the developmental factor PITX2 and the transcription factor Pit-1 [183, 184], although their mechanisms are not yet fully understood.

Recent studies, however, highlighted that, when considering the complexity of regulatory pathways involved in pituitary cell survival and proliferation, it should take into account not only apoptosis but also senescence, an alternative process acting during tumor-suppressive cell fate. Importantly, senescence is gaining biological significance also in pituitary adenomas, whose typical benign nature could result from protective antiproliferative mechanisms. Several transformation events (e.g., DNA damage, loss of tumor-suppressor gene, oncogene activation, and growth factor overexpression) induce preventive cellular senescence, characterized by cell cycle exit and subsequent irreversible proliferation arrest. Thus, pituitary tumors may be more prone to activate senescence-associated pathways, maintaining their benign behavior, preventing malignant transformation, and regulating their development [185]. Interestingly, IL-6 has been shown to participate in oncogene-induced senescence in pituitary gp130 overexpressing tumor cells [96]. Moreover, angiogenic and apoptotic processes cooperate in determining tumor aggressiveness, and this regulation might also be involved in the pathogenesis of pituitary transformation. The pituitary gland is highly vascularized but, unlike other solid malignancies, conflicting results are available on angiogenic factors associated with pituitary adenoma progression and recurrence [186, 187]. VEGF was proposed as pituitary proangiogenic factor and possible therapeutic target [188], and the hypoxia-inducible factor- (HIF-) *α*, a key molecule in hypoxic pathways triggering vessel formation, was detected in pituitary tumor tissues [189, 190] and may favor hemorrhage in pituitary macroadenomas [191].

CKs, particularly CXCL12 signaling* via* CXCR4 and CXCR7, represent candidate mediators of the above described intracellular pathways, determining proliferative, antiapoptotic, and angiogenic signals, thus possibly concurring to pituitary tumor development and aggressiveness.

## 7. Chemokines in Pituitary Tumorigenesis 

Pituitary adenomas are common intracranial tumors of the adenohypophysis causing serious morbidity, due to excessive hormonal secretion, mass effects, and local invasion of surrounding structures. At present, the understanding of biological and molecular pathogenesis and mechanisms of progression of these tumors is largely incomplete (for review see [14, 192, 193]). Emerging evidence reports that multiple factors might contribute to pituitary tumorigenesis, such as frequently altered gene expression, genetic (aryl hydrocarbon receptor interacting protein,* AIP* [194]; multiple endocrine neoplasia syndrome type 1,* MEN1 *[195]; guanine nucleotide-activating alpha subunit,* GNAS, *[196]), and epigenetic (cyclin-dependent kinase inhibitor 2A,* CDKN2A*, or* P16; *FGFR2/melanoma associated antigen -MAGE-3 pathway) [197] mutations, and abnormal microRNAs [198].

The improvement of this knowledge is even more helpful taking into account the peculiar properties of pituitary adenomas as compared to other malignancies: they commonly grow slowly but with local invasive behavior and occasionally develop into high aggressive tumors. This often prevents the efficacy of surgical and systemic medical treatments, the latter hampered by the lack of definite mechanisms underlying pituitary cell transformation and potential therapeutic targets.

The role of CKs in pituitary tumor development has been scantily investigated. However, few studies addressed the potential role of components of this peptide family in regulating human pituitary tumorigenesis.

CXCL8 mRNA was identified in a small percentage of anterior pituitary adenomas [199, 200], altogether with the expression of CXCR2 [201], the CXCL8 receptor that also binds other CXC CKs (CXCL1, CXCL7), confirming the potentiality of autocrine stimulation in pituitary adenomas. Indeed, a consistent release of CXCL8 was observed in primary cultures derived from human somatotroph adenomas, induced by stimulation with interleukin-1*β* and inhibited by GH releasing hormone (GHRH) [202]. Thus, a further assessment of the possible role of this CK in the pathogenesis of pituitary tumors is required, likely being based on CXCL8 ability to recruit active neutrophil within the adenoma, influencing the inflammatory response or acting as mitogen for normal and transformed cells.

However, the majority of studies focused on the analysis of CXCL12 and CXCR4 expression in human neoplastic pituitary tissues and their role in adenoma cell proliferation [128, 129, 203–205].

CXCR4 mRNA is expressed in almost all GH-secreting pituitary adenomas and in the great majority of NFPAs, whilst CXCL12 was identified in about 2/3 of these tumors. Notably, most CXCL12-positive cells also express CXCR4 strongly suggesting an autocrine/paracrine regulation of tumor cell proliferation [129, 205]. This hypothesis was further confirmed measuring the* in vitro* basal secretion of CXCL12 by human pituitary adenoma primary cultures resulting in an autocrine constitutive stimulation of DNA synthesis [129] (Figure 2) and, indirectly, by the absence of CXCR4 activating mutations in GH-secreting and NFPA able to sustain adenoma cell proliferation [204]. This observation was confirmed by a high percentage of different types of secreting pituitary adenomas showing expression of both CXCL12 and CXCR4 [129, 203, 204]. Finally, the evaluation of CXCR4 and CXCL12 expression in invasive and noninvasive pituitary adenoma specimens, by flow cytometry and immunohistochemical staining, demonstrated that the percentage of CXCR4- and CXCL12-positive cells was significantly higher in invasive pituitary adenomas [206]. Thus, the correlation of CXCR4 and CXCL12 expression levels and tumor invasiveness was proposed to be exploited as potential early diagnostic biomarkers, one of the major challenges in diagnosis and treatment of invasive tumors.

Among the mechanisms that occur in pituitary tumorigenesis, angiogenesis represents a key process for tumor growth. Interestingly, while controversial findings on the role of VEGF were reported [207–209], CXCL12 has been proposed as a better defined proangiogenic and proliferative factor in pituitary adenomas. In fact, CXCL12 and CXCR4 are concomitantly upregulated in hypoxic foci within pituitary tumor tissues, and one of the main CXCL12 effects in pituitary adenomas is to mobilize CD34- (and CXCR4-) expressing endothelial progenitors and promote their homing in ischemic foci activating the proangiogenic program [203]. Moreover, in GH3 rat pituitary adenoma cells, hypoxia-activated CXCL12-CXCR4 signaling interacts with the endocrine pathways resulting in upregulation of GH synthesis and secretion and cell proliferation [210]. Thus, in pathological conditions (i.e., hypoxia), on one hand, increased CXCL12 and CXCR4 expression and signalling may promote neoangiogenesis by recruiting endothelial progenitor cells and/or inducing proliferation of endothelial cells and, on the other, directly favouring hormone hypersecretion and pituitary cell proliferation.

Interestingly, GH4C1 rat pituitary adenoma cell line expresses CXCR4 but not CXCL12, thus it was proposed as a suitable model to characterize the molecular pathways regulated by this receptor in pituitary adenomas, without the interference of the endogenously released CK [127, 211, 212]. In these cells CXCL12 exerts a powerful secretagogue and mitogenic activity, as well as promotes cell migration [212]. Interestingly, these effects are induced by different and independent intracellular mechanisms, although all of them were PTX-dependent. GH secretion is a Ca^2+^-dependent event, in which increased ion concentration resulted from IP3-mediated Ca^2+^ release from intracellular stores. Conversely, GH4C1 proliferation is induced by CXCL12 through the activation of ERK1/2 through the “classical” MEK-dependent pathway and* via* the activation of the cytosolic Ca^2+^-dependent tyrosine kinase, Pyk2, that, in turn, activates the large-conductance Ca^2+^-activated K^+^ channels (BKCa) [95, 127, 211, 212]. Similarly, CXCL12/CXCR4 modulation of ERK1/2 activity was reported in GH3 cells [128].

To date, the role of CXCL12/CXCR4 as potential pharmacological target in acromegalic patients has been scantily investigated. It was shown that a synthetic antagonist of CXCR4, d-Arg3FC131, is able to inhibit the growth of GH3 tumor cells and trigger apoptosis both* in vitro* and in mice xenografts [213]. Similar results were obtained using phidianidine A, an indole alkaloid isolated from the marine opisthobranch mollusc* Phidiana militaris*, which reduced GH4C1 proliferation, migration, and ERK1/2 phosphorylation [212].

CXCR7, the second CXCL12 receptor, was reported to be expressed in the AtT20 mouse corticotroph pituitary adenoma cell line [131], but the characterization of its possible role in pituitary adenoma development or progression will require further evaluation.

Studies on human pituitary adenoma cells derived from postsurgical specimens are limited, mostly due to their low proliferative activity* in vitro*. However, such generalized CXCR4 and CXCL12 overexpression in human pituitary adenomas, as compared to normal pituitary [129], strongly suggests that, in conditions of deregulation, this receptor system could be a relevant factor for pituitary adenoma development and/or progression. However, CXCL12 effects of on cell proliferation were directly evaluated* in vitro* on a small number of primary cultures of adenoma cell derived from GHoma, NFPA, and ACTH-secreting adenomas [129]. To avoid interference with the CXCL12 released from fibroblasts a specific protocol to obtain adenoma cell cultures highly purified [214] was used. CXCL12 induced a statistically significant increase in DNA synthesis in the majority (65%) of the adenoma tested. Interestingly, in few adenomas, the blockade of CXCR4 with AMD3100, a known CXCR4 antagonist, caused, beside the abolishment of CXCL12-mediated increase in cell proliferation, also a reduction of basal DNA synthesis. Measuring CXCL12 levels in the culture medium, it was shown that these tumors retained* in vitro* a significant basal secretion of CXCL12 causing a constitutive, autocrine stimulation of DNA synthesis [129]. Thus, these data suggest that pituitary adenoma cells over-express CXCL12 and CXCR4 as compared to normal tissue and that an autocrine activation of this pathway (Figure 2), actually, occurs* in vivo*.

Overall, these observations imply that CXCL12/CXCR4 axis might play an important biological role in pituitary adenoma as potential growth and angiogenic factor for pituitary cells. The increased CXCR4 activation may result from either endocrine (increased CXCL12 levels may reach pituitary through the blood stream or being released in the portal pituitary system from hypothalamus) and/or autocrine/paracrine mechanisms (Figure 2). Importantly, the latter mechanism seems to be active mainly in pituitary tumors rather than in normal gland, where most of the CXCR4-expressing normal cells do not express CXCL12 [129].

## 8. Conclusions 

Chemokines are key factors in CNS physiology and pathology, being relevant mediators in cancer development. The CXCL12/CXCR4-R7 signaling pathway plays a unique role in the regulation of a variety of cell types, including embryonic and cancer cells. In particular, deregulation of this chemokinergic system is strictly related to tumor initiation and progression, and the balance of its activity within the tumor microenvironment is highly complex phenomenon.

Multiple factors cooperate to pituitary tumor pathogenesis, but, although to date not explored in depth, a pivotal role of CK and their receptors seems to be important. Several mechanisms by which CXCL12/CXCR4 modulates pituitary function and promotes adenoma cell proliferation and their target as potential therapeutic approach have been suggested. CXCL12 is overexpressed in pituitary cells where it can directly influence adenoma formation, through autocrine mechanisms resulting in constitutive CXCR4 activation. This pathway grants pituitary cells with a proliferative advantage that triggers clonal expansion of transformed cells and sustains tumor cell survival. Increasing knowledge about pituitary cell origin and development might provide significant insights into deregulated pathways in pituitary tumorigenesis.

Finally, CXCR4 is an easily druggable target and the characterization of its role in pituitary adenomas could pave the way for novel pharmacological approaches, especially for those adenoma subtypes, (i.e., TSH and ACTH secreting tumors, as well as NFPA) still waiting for efficacious drugs.
